# Pregnancy Outcomes of Non-Visualization of the Fetal Gallbladder from a Chinese Tertiary Single Centre and Literature Review

**DOI:** 10.3390/children9091288

**Published:** 2022-08-26

**Authors:** Huijing Zhang, Xiaoying Zhu, Jinling Kang, Yu Sun, Huixia Yang

**Affiliations:** 1 Department of Obstetrics and Gyanaecology, Peking University First Hospital, Beijing 100034, China; 2 Department of Ultrasound, Hengshui Renmin Hospital, Hengshui 053000, China; 3 Department of Ultrasound, Dezhou Women and Children’s Hospital, Dezhou 251500, China

**Keywords:** non-visualization of the fetal gallbladder (NVFGB), pregnancy outcomes, prognosis

## Abstract

Objection: To explore the clinical features and prognosis of non-visualization of the fetal gallbladder (NVFGB). Methods: Sixty-five cases diagnosed with NVFGB in the Peking University First Hospital were collected retrospectively from January 2019 to December 2020. Results: Forty-nine cases were successfully followed up. Among them, the gallbladder of 21 fetuses (42.9%) was present in the later pregnancy. In the rest 28 cases (57.1%), the gallbladders were absent during the whole pregnancy. Eleven of twenty-eight fetuses (39.3%) with NVFGB were complicated with other structural anomalies. In the remaining 17 cases of isolated NVFGB (60.7%) during the whole pregnancy, there was one case of congenital biliary atresia, three cases of a small gallbladder, one case of gallstone and one case of the gallbladder with several septa inside. A total of nine cases (18.4%) underwent prenatal diagnosis, four of which revealed abnormal copy number variant (CNV) results. Conclusion: Nearly half of NVFGB could be noted during the later pregnancy. The persistent NVFGB during the pregnancy has a higher risk to complicate gallbladder abnormality, other structural anomalies and abnormal CNV results. Other structures, especially the heart, gastrointestinal and urinary system, should be carefully examined when NVFGB is suspected.

## 1. Introduction

The gallbladder origins from the hepatic diverticulum on the ventral side of the primitive midgut in the fourth week of embryonic development and begins cavitation into a cystic structure in the twelfth week [1]. Therefore, theoretically, the morphological structure of the gallbladder can be viewed by prenatal ultrasound from the first trimester of pregnancy. In 1987, Hata et al. described ultrasonic characteristics of the fetal gallbladder for the first time. They found that the gallbladder detection rates at 20–23 gestational weeks and at 24–27 gestational weeks were 37.5% and 64.7%, respectively [2]. Some studies reported a high detection rate of 99.9% at 14–16 weeks [3,4] by transvaginal ultrasonography, while 95% at 24–32 weeks of pregnancy by transabdominal ultrasonography [5].

Non-visualization of the fetal gallbladder (NVFGB) refers to the inability to observe the fetal gallbladder in two or more consecutive ultrasound examinations within one week during pregnancy. The prevalence of NVFGB was 0.1–0.15% [3,4]. In most cases, the fetal gallbladder is noted during the following prenatal ultrasound or postnatal ultrasound examinations. However, Dreux et al. revealed that NVFGB was associated with gallbladder dysplasia, biliary atresia, cystic fibrosis, chromosome anomalies and intestinal obstruction [6].

The purpose of this study was to summarize and discuss the clinical characteristics, treatment and prognosis of NVFGB in the Chinese population.

## 2. Data and methods

### 2.1. Study Subjects

In this study, clinical data of 65 fetuses who were initially diagnosed with NVFGB at the Department of Obstetrics and Gynaecology, Peking University First Hospital, from January 2019 to December 2020 were collected retrospectively. Among them, 16 patients failed to follow up, including 1 case of late inevitable abortion and 15 cases of refusal to follow up.

### 2.2. Instruments and Study Methods

#### 2.2.1. Instruments and Ethic Statement

All scans were performed by certified radiologists with at least five years of experience in fetal ultrasound. We received all the written consent forms from these patients. The study was conducted according to the guidelines of the Declaration of Helsinki and approved by the Institutional Review Board of Peking University First Hospital (protocol code 2013[572]).

#### 2.2.2. Methods to Examine Foetal Gallbladder

**Figure 1:**
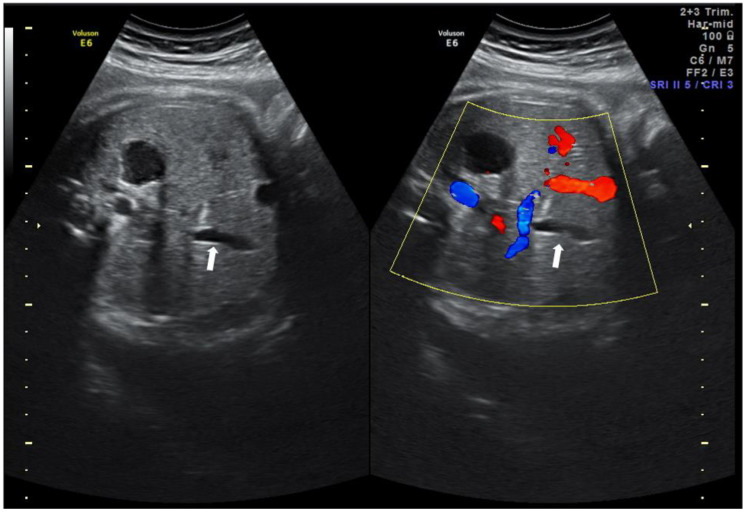
Image of the fetal gallbladder (white arrow) noted using 2D ultrasound with and without color.

During the ultrasound, the view of the gallbladder could be seen at the abdominal circumference plane. It was generally on the right side umbilical vein [7]. In two-dimensional (2D) imaging, the gallbladder was a hypoechoic area with a hyperechoic cystic wall (Figure 1). The shape varies, including teardrop, rectangle, spindle, annulus, etc. [8]. Colour Doppler ultrasound was used to distinguish the gallbladder from the intra-abdominal segment of the umbilical vein. NVFGB was diagnosed if no gallbladder was observed in 2 or more ultrasound examinations within one week.

#### 2.2.3. Routine Ultrasonic Examinations

The mid-trimester fetal anomaly scan was performed between 20 and 23^+6^ gestational weeks during our clinical practice. If NVFGB was suspected, the other fetal anomalies were further examined in detail according to the ultrasonic examination guidelines by the International Society of Ultrasound in Obstetrics and Gynaecology (ISUOG) [9]. Our center provides prenatal consultation and prenatal diagnosis for pregnant women complicated with fetal structural anomalies. The patient and her family decided on whether to conduct an invasive prenatal diagnosis.

#### 2.2.4. Data Collection

The relevant clinical data were collected for all patients, including age, gravidity and parity history, method of fertilization, gestational age at the initial finding of NVFGB, other fetal structural anomalies, pregnancy outcomes, gestational age at birth, birth weight and follow-up after birth, etc. Telephone follow-up was conducted after birth. The last follow-up occurred on 25 January 2021.

#### 2.2.5. Statistical Analysis

A descriptive analysis was conducted using the clinical data of NVFGB cases. The mean (±standard deviation) was calculated for the counting variable, and the number of cases (percentage) was reported for the categorical variable.

## 3. Results

### 3.1. General Data

The average age of the 49 pregnant women included in this study was 30.78 years, and the mean gestational age at which NVFGB was initially observed was 26.22 weeks (Table 1).

### 3.2. Pregnancy Outcomes of NVFGB (Table 2)

In 21 cases of temporary NVFGB (21/49, 42.86%), the gallbladder was noted during the late pregnancy. In the other 28 cases (57.1%), the gallbladders were absent during the whole pregnancy. Among the 17 cases of isolated NVFGB (17/28, 60.7%), one case was diagnosed with type III congenital biliary atresia after birth and underwent surgical treatment. The other five abnormal cases contained three cases of the small gallbladder, one case of gallstones and one case of the gallbladder with several septa inside. The gallbladder function of these infants was normal after birth with no interventions. The gallbladder of the remaining 10 cases (59%) was noted after birth.

**Table 2:** Pregnancy outcome of the temporary and persistent NVFGB.

Items	Temporary NVFGB (*n* = 21)	Persistent NVFGB (*n* = 28)
Isolated	18 (85.7%)	17 (60.7%)
Non-gallbladder anomalies	3 (14.3%)	11 (39.3%)
Details	VSD Hydronephrosis Bowel atresia	TOF, Truncus Arteriosuss, VSD, Aortic stenosis Horseshoe kidney, ovarian cyst Anal atresia Open spina bifida,
Outcomes		
Live birth	21 (100%)	20 (71.4%)
IUD	0	1 (3.6%)
TOP	0	7 (25%)
Gallbladder outcome		
Abnormality	0 (0)	6 (21.4%)

TOF, Tetralogy of Fallot; VSD, ventricle septum defect; IUD, intrauterine death; TOP, termination of pregnancy.

### 3.3. Analysis of the Prenatal Diagnosis of NVFGB

In this study, nine patients agreed to undergo amniocentesis, whose indication was three cases of multiple anomalies, two cases of advanced maternal age (≥35 years old) and three cases of persistent NVFGB, respectively. Consequently, the result of four cases was abnormal and persistent NVFGB was noted in the three cases, as shown in Table 3.

## 4. Discussion

The incidence of NVFGB during pregnancy is low. According to the data from our center, the incidence of NVFGB was 0.4% (49/12,049), and the percentage of persistent NVFGB was 0.2% (28/12,049), which was similar to the previously published result [3,4]. The reason for the slightly higher incidence was probably because our institute was a third-tertiary care center responsible for patient referrals with suspected fetal anomalies from other regions. Most of the relevant articles were case series reports [3,8,10,11,12]. Blazer et al. detected 34 foetuses of NVFGB among 29,749 pregnancies (0.1%) [3]. In the study by Bardin et al., the amniotic fluid levels of gamma-glutamyl-transpeptidase (GGTP) in 32 cases of NVFGB were analyzed [8]. In addition, Ochshorn et al. reported 22 cases of isolated NVFGB [10]. Shen et al. collected 21 cases of NVFGB [11], while Sagi-Dain et al. examined the frequency of abnormal genetic results among 45 cases of isolated NVFGB [12].

It is indicated that most NVFGB during pregnancy is temporary. In Di Pasquo et al.’s study, 75% of fetal gallbladders were noted during late pregnancy [13]. In our study, 42.86% (21/49) of fetal gallbladders were present afterward. The percentage was slightly lower than that in the literature, possibly due to the early termination of pregnancy in some cases. The temporarily invisible gallbladder in prenatal ultrasound might be associated with the temporary contraction of the gallbladder [14].

In a multicentre retrospective study, 7.8% of cases were diagnosed with congenital biliary atresia, of whom 5.9% were not complicated with other structural anomalies [6]. Among the fetuses with isolated NVFGB in this study, only one (4%) was diagnosed with biliary atresia after birth. The infant underwent the Kasai procedure 40 days after birth; however, the outcome after the operation was poor. Therefore, liver transplantation was performed three months after birth. The infant is now in good condition. Congenital biliary atresia is a severe neonatal disease with an incidence of 0.5–0.8/10,000 live births in Western countries [15,16] and a relatively high incidence of 1.1–1.5/10,000 live births in Asian countries [17,18]. According to the location of atresia, biliary atresia can be divided into type I (5%, atresia located at the level of the common bile duct), type II (2%, atresia located at the level of the common hepatic duct) and type III (>90%, atresia located at the level of the hepatic portal vein). The typical ultrasonic feature is an absent gallbladder or small gallbladder. If the disease is not identified and treated promptly, it leads to severe complications and even death [19]. The Kasai procedure can significantly improve the prognosis of newborns. The best timing of the operation is within 60 days after birth, resulting in a 10-year survival rate as high as 70% [20]. Liver transplantation can be performed if the surgical outcome is poor. As reported in the literature, the 1-year survival rate and the 3-year survival rate after liver transplantation are 90% and 88%, respectively [21].

NVFGB is an ultrasound finding of cystic fibrosis (CF) during pregnancy [6,22,23]. However, in this study, CF was not discovered, which may be related to the ethnicity of the study population. Cystic fibrosis has high incidence rates in Europe, North America and Australia. It is the most common fatal genetic disease leading to lung diseases, digestive system obstruction, pancreatic dysplasia, infertility, etc. [24]. It is caused by a mutation in the cystic fibrosis transmembrane conductance regulator (CFTR) gene [25]. The protein encoded by CFTR is involved in regulating sodium/chlorine channels in epithelial cells [26]. The main prenatal ultrasound manifestations are fetal digestive system anomalies, such as hyperechogenic bowel, dilated bowel and NVFGB. Becdelièvre A et al. indicated that NVFGB, together with hyperechogenic and dilated bowel has a high predictive value for the prenatal diagnosis of cystic fibrosis (likelihood ratio, 31.4) [22]. Bergougnoux et al. summarized the prenatal ultrasound features of 37 fetuses with cystic fibrosis. Among them, 5 (13.5%) cases had isolated NVFGB [27].

In our study, only nine fetuses underwent invasive prenatal testing during pregnancy, and four of nine were found to have abnormal results. The absence of a gallbladder was related to chromosomal anomalies. The most common chromosomal anomalies include trisomy 8p [28], partial trisomy 16q [29], trisomy 16 [30], trisomy 22 [31], trisomy 47 [32], etc. However, in those studies, fetuses were complicated with other ultrasonic structural anomalies. In studies of the prenatal Diagnosis of isolated NVFGB, Bronstein et al. [4] and Blazer et al. [3] did not find chromosome anomalies in the isolated NVFGB. Ochshorn et al. demonstrated only one case of 47, XXX [10]. Lena et al. reported one pathological chromosomal microarray analysis (CMA), but its relationship with gallbladder development was uncertain; the other two abnormal CMA results had no clear clinical significance, which was consistent with our results (Table 3). The incidence of abnormal CMA results was not significantly different from that in the normal population [12]. There are few studies regarding the abnormal development of the foetal gallbladder and abnormal chromosome genes. The clinical significance of genetic anomalies in published articles is still unclear. It is necessary to collect further data for prenatal consultations.

However, the limitation of this study is that, firstly. It is a retrospective study based on data from a single tertiary center, resulting in small numbers of abnormal cases and bias. Secondly, the genetic results were obtained from only nine fetuses. Last but not least, the follow-up should be continued in the future.

In conclusion, nearly half of NVFGB could be noted during later pregnancy. The persistent NVFGB during the pregnancy has a higher risk to complicate gallbladder abnormality, other structural anomalies and abnormal CNV results. Other structures, especially the heart, gastrointestinal and urinary system, should be carefully examined when NVFGB is suspected. Additionally, patients with isolated NVFGB during pregnancy should be informed that there is still a possibility (3–6%) of a congenital biliary atresia diagnosis after birth and that the prognosis of patients after the operation is good [13].

## Figures and Tables

**Table 1:** Demographic data and clinical characteristics of the study population.

Clinical Data	Value
Age	30.78 (±4.19)
Mode of conception	
Natural pregnancy	45 (91.8%)
Assisted reproductive technology	4 (8.2%)
Singleton	47 (95.9%)
Twins *	2 (4.1%)
Gestational age at initial finding of NVFGB	26.22 (±4.72)
Fetal outcomes	
Live birth	41 (83.7%)
Intrauterine death (IUD)	1 (2%)
Cholestasis and heart PLSVC	1
Termination of pregnancy (TOP)	7 (14.3%)
Tetralogy of Fallot (TOF)	2
Truncus arteriosus and VSD and SUA	1
Aortic stenosis and Horse shoe kidney	1
VSD and FGR	1
Open spina bifida	1
Persistent NVFGB	1

* In both twin pregnancies, only one of the twins had NVFGB. IUD, intrauterine death; PLSVC, persistent left superior vena cava; TOP, termination of pregnancy; TOF, Tetralogy of Fallot; VSD, ventricle septum defect; SUA, single umbilical artery; FGR, foetal growth restriction.

**Table 3:** Abnormal genetic results of NVFGB who underwent prenatal diagnosis.

Case	Genetic Results	Ultrasonic Feature during Pregnancy	Pregnancy Outcome
1	Arr [hg19] 18q21.1 (44396913-44701650) x1, deletion, VUS	Temporary NVFGB complicated with a ventricular septal defect	Live birth, ventricular septal defect repair
2	Arr [hg19] 5p14.3 (21529026-22296824) x1, deletion, VUS	Isolated persistent NVFGB during pregnancy	Live birth, type 3 congenital biliary atresia
3	Arr [GRCh37] 2q22.1q22.2 (141798723_42726446) x1, deletion, VUS	Isolated persistent NVFGB during pregnancy	Live birth, generally good condition, paternal origin
4	47, XX + 9[44]/46, XX [34]	Persistent NVFGB complicated with open spina bifida	TOP

## Data Availability

The data presented in this study are available on request from the corresponding author.

## References

[1] Ando H. (2010). Embryology of the Biliary Tract. Dig. Surg..

[2] Hata K., Aoki S., Hata T., Murao F. (1987). Ultrasonographic Identification of the Human Fetal Gallbladder in utero. Gynecol. Obstet. Investig..

[3] Blazer S., Zimmer E.Z., Bronshtein M. (2002). Nonvisualization of the fetal gallbladder in early pregnancy: Comparison with clinical outcome. Radiology.

[4] Bronshtein M., Weiner Z., Abramovici H., Filmar S., Erlik Y., Blumenfeld Z. (1993). Prenatal diagnosis of gall bladder anomalies—Report of 17 cases. Prenat. Diagn..

[5] Hertzberg B.S., Kliewer M.A., Maynor C., McNally P.J., Bowie J.D., Kay H.H., Hage M.L., Livingston E. (1996). Nonvisualization of the fetal gallbladder: Frequency and prognostic importance. Radiology.

[6] Dreux S., Boughanim M., Lepinard C., Guichet A., Rival J.M., de Becdelievre A., Dugueperoux I., Muller F. (2012). Relationship of non-visualization of the fetal gallbladder and amniotic fluid digestive enzymes analysis to outcome. Prenat. Diagn..

[7] Wang Y., Li S., Chen Z. (2011). Prenatal Ultrasound diagnosis of fetal gallbladder anomalies. Chin. J. Med. Ultrasound.

[8] Bardin R. (2016). Nonvisualization of the Fetal Gallbladder: Can Levels of Gamma-Glutamyl Transpeptidase in Amniotic Fluid Predict Fetal Prognosis?. Fetal Diagn. Ther..

[9] Salomon L.J., Alfirevic Z., Berghella V., Bilardo C., Hernandez-Anade E., Johnsen S.L., Kalache K., Leung K.Y., Malinger G., Munoz H. (2011). Practice guidelines for performance of the routine mid-trimester fetal ultrasound scan. Ultrasound Obstet. Gynecol..

[10] Ochshorn Y., Rosner G., Barel D., Bronshtein M., Muller F., Yaron Y. (2007). Clinical evaluation of isolated nonvisualized fetal gallbladder. Prenat. Diagn..

[11] Shen O., Rabinowitz R., Yagel S., Gal M. (2011). Absent gallbladder on fetal ultrasound: Prenatal findings and postnatal outcome. Ultrasound Obstet. Gynecol. Off. J. Int. Soc. Ultrasound Obstet. Gynecol..

[12] Sagi-Dain L., Singer A., Hadid Y., Sharony R., Vinkler C., Bar-Shira A., Segel R., Ben Shachar S., Maya I. (2019). Non-visualization of fetal gallbladder in microarray era-a retrospective cohort study and review of the literature. J. Matern.-Fetal Neonatal Med. Off. J. Eur. Assoc. Perinat. Med. Fed. Asia Ocean. Perinat. Soc. Int. Soc. Perinat. Obs..

[13] Di Pasquo E., Kuleva M., Rousseau A., Vitucci A., Sonigo P., Chardot C., Salomon L.J., Ville Y. (2019). Outcome of non-visualization of fetal gallbladder on second-trimester ultrasound: Cohort study and systematic review of literature. Ultrasound Obstet. Gynecol. Off. J. Int. Soc. Ultrasound Obstet. Gynecol..

[14] Wang Y., Li S., Chen Z., Wen H., Yao Y., Bi J., Liao Y. (2012). Diagnosis and clinical evaluation of gallbladder anomalies by prenatal ultrasound screening. Chin. J. Med. Ultrasound.

[15] Hartley J.L., Davenport M., Kelly D.A. (2009). Biliary atresia. Lancet.

[16] Nizery L., Chardot C., Sissaoui S., Capito C., Henrion-Caude A., Debray D., Girard M. (2016). Biliary atresia: Clinical advances and perspectives. Clin. Res. Hepatol. Gastroenterol..

[17] Chiu C.Y., Chen P.H., Chan C.F., Chang M.H., Wu T.C. (2013). Biliary atresia in preterm infants in Taiwan: A nationwide survey. J. Pediatr..

[18] Wada H., Muraji T., Yokoi A., Okamoto T., Sato S., Takamizawa S., Tsugawa J., Nishijima E. (2007). Insignificant seasonal and geographical variation in incidence of biliary atresia in Japan: A regional survey of over 20 years. J. Pediatric Surg..

[19] Sanchez-Valle A., Kassira N., Varela V.C., Radu S.C., Paidas C., Kirby R.S. (2017). Biliary Atresia: Epidemiology, Genetics, Clinical Update, and Public Health Perspective. Adv. Pediatrics.

[20] Ohi R., Nio M., Chiba T., Endo N., Goto M., Ibrahim M. (1990). Long-term follow-up after surgery for patients with biliary atresia. J. Pediatric Surg..

[21] Utterson E.C., Shepherd R.W., Sokol R.J., Bucuvalas J., Magee J.C., McDiarmid S.V., Anand R. (2005). Biliary atresia: Clinical profiles, risk factors, and outcomes of 755 patients listed for liver transplantation. J. Pediatr..

[22] de Becdelièvre A., Costa C., Jouannic J.M., LeFloch A., Giurgea I., Martin J., Médina R., Boissier B., Gameiro C., Muller F. (2011). Comprehensive description of CFTR genotypes and ultrasound patterns in 694 cases of fetal bowel anomalies: A revised strategy. Hum. Genet..

[23] Duguépéroux I., Scotet V., Audrézet M.P., Saliou A.H., Collet M., Blayau M., Schmitt S., Kitzis A., Fresquet F., Müller F. (2012). Nonvisualization of fetal gallbladder increases the risk of cystic fibrosis. Prenat. Diagn..

[24] Elborn J.S. (2016). Cystic fibrosis. Lancet.

[25] Riordan J.R., Rommens J.M., Kerem B., Alon N., Rozmahel R., Grzelczak Z., Zielenski J., Lok S., Plavsic N., Chou J.L. (1989). Identification of the cystic fibrosis gene: Cloning and characterization of complementary DNA. Science.

[26] Welsh M.J., Smith A.E. (1993). Molecular mechanisms of CFTR chloride channel dysfunction in cystic fibrosis. Cell.

[27] Bergougnoux A., Jouannic J.M., Verneau F., Bienvenu T., Gaitch N., Raynal C., Girodon E. (2019). Isolated Nonvisualization of the Fetal Gallbladder Should Be Considered for the Prenatal Diagnosis of Cystic Fibrosis. Fetal Diagn. Ther..

[28] Fryns J.P., Petit P., Moerman F., Cassiman J.J., van den Berghe H. (1982). 8p trisomy in a malformed foetus. Ann. Genet..

[29] Hatanaka K., Ozaki M., Suzuki M., Murata R., Fujita H. (1984). Trisomy 16q13—qter in a infant from a t(11;16)(q25;q13) translocation-carrier father. Hum. Genet..

[30] Moradkhani K., Puechberty J., Blanchet P., Coubes C., Lallaoui H., Lewin P., Lefort G., Sarda P. (2006). Mosaic trisomy 16 in a fetus: The complex relationship between phenotype and genetic mechanisms. Prenat. Diagn..

[31] Gangbo E., Lacombe D., Alberti E.M., Taine L., Saura R., Carles D. (2004). Trisomy 22 with thyroid isthmus agenesis and absent gall bladder. Genet. Couns..

[32] Matheson J.K., Matheson V.A., McCorquodale M., Santolaya-Forgas J. (2003). Prenatal diagnosis of double autosomal mosaicism (47,XX,+8/47,XX,+14): Phenotype and molecular cytogenetic analysis on different tissues. Fetal Diagn. Ther..

